# Beyond awareness: the binding of reflexive mechanisms with the conscious mind: a perspective from default space theory

**DOI:** 10.3389/fnhum.2024.1520138

**Published:** 2024-12-12

**Authors:** Ravinder Jerath, Connor Beveridge

**Affiliations:** Charitable Medical Healthcare Foundation, Augusta, GA, United States

**Keywords:** reflexes, multisensory integration, consciousness, default space, phenomenology, top-down modulation

## Abstract

How do reflexes operate so quickly with so much multimodal information on the environment? How might unconscious processes help reveal the nature of consciousness? The Default Space Theory of Consciousness (DST) offers a novel way to interpret these questions by describing how sensory inputs, cognitive functions, emotional states, and unconscious processes are integrated by a single unified internal representation. Recent developments in neuroimaging and electrophysiology, such as fMRI, EEG, and MEG, have improved our knowledge of the brain mechanisms that underpin the conscious mind and have highlighted the importance of neural oscillations and sensory integration in its formation. In this article, we put forth a perspective on an underresearched relationship of reflexes with the dynamic character of consciousness and suggest that future research should focus on the interplay of the unconscious processes of reflexes and correlates of the contents of consciousness to better understand its nature. Existing research on the top-down cortical influence over the subcortical operations of reflexes is severely lacking. This top-down influence has been demonstrated, but how the complex multimodal model of the self and environment is encoded and utilized to produce quick and coordinated reflex responses is not understood. Integrating unconscious/subconscious reflexive mechanisms with models of consciousness may illuminate a boundary between or gradient among conscious and unconscious activity. This perspective in light of the DST’s framework may reveal future research avenues aimed at understanding the complexities and physical nature of consciousness.

## Introduction

While there are many approaches to modeling consciousness, this academic field is still in its early stages due to the sheer difficulty of the problem and of empirically studying it. A prominent category of consciousness theories are the electromagnetic theories which propose that consciousness is isomorphic to certain electromagnetic phenomenon of the nervous system (MacIver, 2022). These theories provide an effective solution to the binding problem, the problem of how the vast distributed information throughout the brain is integrated into a single experience (Revonsuo and Newman, 1999). A unified bioelectric architecture would allow the integration of this distributed neuronal information evident in consciousness. Several electromagnetic field theories exist including the electromagnetic field theory proposed by Pockett (2012), the CEMI field theory by McFadden (2013, 2020), the Operational Architectonics theory by Fingelkurts and Fingelkurts (2001) and Fingelkurts et al. (2009), and the Default Space Theory (DST) by Jerath and Beveridge (2018, 2019) and Jerath et al. (2015a).

The DST is unique from these theories largely in the fact that it includes the peripheral nervous system in the bioelectric architecture proposed to be isomorphic to consciousness (Jerath et al., 2019). According to the DST, consciousness is not merely a passive recipient of sensory input but rather an active and dynamic process that is centered upon maintaining an internal representation of both the external environment and the self (Jerath et al., 2015a). This idea that our experience of the world is fundamentally shaped by our bodily form and the environment was also proposed early on by embodied or grounded theories of consciousness (Barsalou, 2010). According to these theories, the conscious mind emerges from the interactions between the body, brain, and environment (Varela et al., 1991). Perceptual experiences are thus not just passive reception of sensory data, but an embodied engagement with the world (Baldwin, 2003). More modern versions of these theories argue that body and environment-related representations coordinate and contextualize global brain function (Allen and Friston, 2018).

**FIGURE 1:**
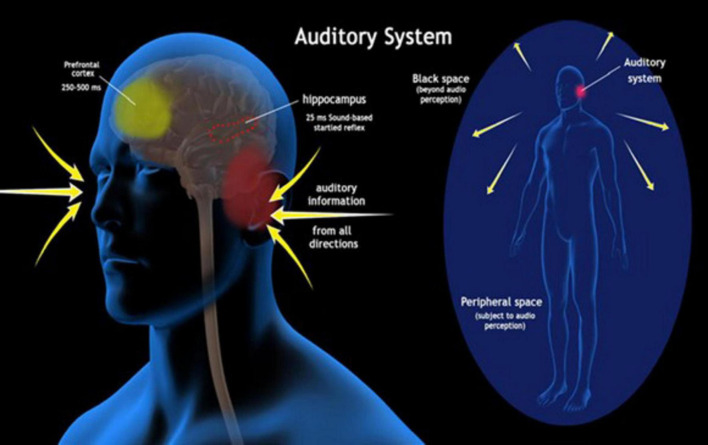
The auditory system: The image illustrates the auditory system with regards to conscious and unconscious processing. Incoming sound waves, represented by yellow arrows, are received from all directions around the body. The peripheral space, shown as a blue oval surrounding the human figure, represents the area subject to audio perception by the auditory system, while the “Black space” area represents regions beyond the range of audio perception. The hippocampus itself is highlighted as a key brain region involved in auditory processing for sound-triggered startle reflexes occurring within 25 ms. The prefrontal cortex, with a processing time of 250–500 ms, is involved in higher-order cognitive processing of auditory information.

Reflexes are an automatic first line of defense against major and minor impending dangers. For reflexes to effectively guide us away from such dangers, they must somehow utilize information about the environment (Sambo et al., 2012). Sensory information utilized by unconscious reflexes undergoes a different route of processing than when being integrated into consciousness. Most reflexes are subserved by subcortical circuits at the brainstem level and modulated by top-down influences from the cerebellum and cortical areas (Wallwork et al., 2016). On the other hand, sensory information entering consciousness is largely percolated throughout the thalamocortical system (Olcese et al., 2018). Reflexes operate at significantly faster speeds than the half-second delay required for sensory information to enter consciousness (Libet et al., 1979). In the auditory system for instance (Figure 1), auditory signals entering consciousness make their way through the brainstem and thalamus up to the auditory cortex (Munkong and Juang, 2008) and also to the prefrontal cortex for advanced auditory cognition (Plakke and Romanski, 2014). When utilized in reflexes, acoustic information is largely processed in the caudal pontine reticular nucleus of the brainstem (Lee et al., 1996) and modulated by the limbic system including the amygdala, hippocampus, and stria terminalis (Lee and Davis, 1997).

The circuitry involved in the functionality of reflexes and their dependence on top-down inputs are not yet fully understood. There is still much research needed to reveal how reflexes operate so quickly and effectively with so much information about the environment (Valls-Solé and Hallett, 2019). They should be regarded as integrated actions that may be modulated by higher centers of the cortex to respond appropriately to a fast-changing environment (Sherrington, 1906). In this article, we put forth a perspective that lower-level processes of reflexes and potentially other unconscious processes are unified with a global bioelectric structure that is isomorphic to a representation of the internal and external environment. In the delivery of our perspective, we focus on the defensive reflexes which purposefully utilize higher-order cognition, particularly the complex multimodal conscious representation of the environment and self which is the contents of consciousness. These reflexes exist more at the boundary of consciousness. Reflexes like the baroreceptor reflex exist far beyond the boundary of consciousness and so likely will not be helpful in delineating such a boundary. We hope our perspective will inspire researchers to investigate the nature of top-down influence in the production of reflexes and that it may reveal a further means to differentiate unconscious, conscious, and subconscious neural activity.

## Neural and phenomenological foundations of consciousness

The term “Default Space” of the DST refers to one of the theory’s central tenets, that the foundation of the cognitive and phenomenological mind is a virtual, three-dimensional coordinate matrix in which all qualia are embedded (Jerath and Beveridge, 2019). The perspective that a three-dimensional space is the most fundamental aspect of human experience is shared among many preeminent and founding consciousness theorists and researchers (Damasio, 1999; Fingelkurts et al., 2010; Revonsuo, 2006). We all experience the world from the mathematical origin of this virtual space which is a direct replication of the universe in which we live (Revonsuo, 2006; Siegel, 2006) and provides a localizing sense of self (Blanke and Metzinger, 2009; Metzinger, 2003; Trehub, 2007). The DST proposes the default space, both a cognitive and phenomenological entity, is manifested biologically from a cohesive, multi-modal, oscillatory bioelectric architecture present across the central and peripheral nervous system (Jerath et al., 2019).

According to the DST, the global oscillatory architecture isomorphic to consciousness serves not only as a foundation of cognitive and phenomenal structures but includes a unified integration of sensory organs (Jerath et al., 2019). For instance, the process of vision involves a complex interplay between the retina and various regions of the brain (including the brainstem, thalamus, and higher cortical areas), linked not only via bottom-up neural signaling but by bi-directional unification via oscillatory activity (Castelo-Branco et al., 1998; Jerath et al., 2016; Neuenschwander et al., 2002). The multimodal integration of sensory inputs facilitates a comprehensive representation of the external environment and self. The DST and several other theories posit the thalamocortical system as the functional hub of global module integration (Baars et al., 2013; Jerath et al., 2019; Tononi and Edelman, 1998) which would allow for the architecture for such a global oscillatory structure.

A body-wide bioelectric architecture satisfies the requirement of global, binding coherence for a coalesced consciousness, synchronizing local computations in widespread symphony (Jerath and Beveridge, 2019). The oscillatory symphony of consciousness is mediated by the interconnectedness of several regions in the brain, with each region playing a unique role in its formation. The thalamus along with areas of the brainstem act as the “conductor” of this symphony, orchestrating the many regions of the brain (Jerath et al., 2015a). Syndromes like Contralateral Neglect Syndrome and Phantom Limb Syndrome also point to the existence of a continuously maintained neural representation of the environment and self that spans the conscious and unconscious mind. Disruptions to neural circuits that maintain this representation, whether through injury or deafferentation (loss of sensory input), can lead to distortions or even hallucinations in our perception of the world (Jerath and Crawford, 2014; Jerath et al., 2015b; Ramachandran and Blakeslee, 1999).

## The binding of reflexive mechanisms with the contents of consciousness

Individuals navigate in a fast-changing environment, and so defensive responses must adjust rapidly as a function of the predicted spatial position and nature of external threats (Wallwork et al., 2016). Reflexes process utilize and initiate responses to sensory information before it enters conscious awareness. The fastest human reflexes like the blink reflex take around 20 ms and slower reflexes such as those utilizing the legs still respond in about 150 ms (Davis, 1984). Some startle reflexes in animals like the rat occur within 8 ms indicating only a few synapses being involved in the startle circuit (Davis et al., 1982). Reflex mechanisms of startle and other defensive reflex circuits, however, often utilize complex multimodal sensory and cognitive information in producing an appropriate response, for instance, information indicating the location and type of potential threat (Wallwork et al., 2016). For this sort of speed to occur in defensive reflexes with such a sensory and cognitively mediated action, the unconscious neural processes underlying reflexes must be integrated or united with some sort of cognitive simulation of the environment which so defines our phenomenal mind. This would bypass a need for separate time-consuming computation on vast amounts of afferent sensory data to determine any potential reflexive needs given dangers in the environment.

Spatial information and other aspects of potential threats are utilized in producing a defensive reflexive response. There is a portion of space surrounding the face, defensive peripersonal space (DPPS), with a protective cognitive attention. Potential threats occurring within this space elicit stronger defensive responses compared to those located outside of it (Bufacchi et al., 2016; Graziano and Cooke, 2006; Sambo et al., 2012). Neural processes underlying startle reflexes link the blink reflex are indeed able to exploit predictions on the spatial location of threatening stimuli in a deliberate and purposeful manner with both the current and future predicted positions of the potential threat in respect to the body being accounted for. The environmental information about potential threats are exploited via top-down modulation from the cortex to the brainstem. This top-down modulation is continuously and purposefully administered (Wallwork et al., 2016). Its nature is determined by a number of additional high-level cognitive factors. For example, the estimated probability that the threatening stimulus will occur, as well as on the presence of defensive objects (Sambo et al., 2012).

Attention unconsciously generated by reflexive processes acts as a filter, directing our focus on specific aspects of the sensory environment. The integration of reflexes with the conscious mind is further evident through priming (unconscious reflexes influencing what captures conscious attention), habituation (repeated stimuli triggering a decrease in the reflex response that conscious attention can override), and voluntary control (exerting conscious control over reflexes in some cases) (Barrett et al., 2004; Kiefer, 2008; Zeman, 2001). The integration of lower brainstem reflexive processes with higher-order cortical ones is thus bidirectional in influence. Such a bi-directional interface between the contents and structure of the phenomenal mind and the coordinated actions of reflexes suggests the existence of a global cognitive architecture isomorphic to the phenomenal representation of the self and environment.

## Discussion

Imagine a scenario in which someone has thrown a ball to you while you are not looking. You are aware of a ball in the environment and they yell at you to look out. Your brain utilizes the knowledge of the existence of the ball, of the spatial environment (including self-position), and the direction of the sound to produce a reflex to dodge the ball just in time. For such defensive reflexes dependent on multisensory input to operate successfully at such fast speeds, they must utilize such an active, pre-existing model of the self and environment. Defensive reflexes and conscious activities must be acting at different levels or scopes of neural processing but are, however, necessarily linked. Observations on the apparent integration of these reflexive processes with the contents of consciousness suggest that the unconscious neural processes of reflexes are integrated at a fundamental level with a global cognitive entity isomorphic to the phenomenal mind.

An incorporation of these unconscious activities into cognitive models of mind may help achieve a more comprehensive understanding of consciousness and its correlates. The boundary between conscious and unconscious activity may be identified by investigating isomorphic systems at this boundary, for instance between the brainstem-based startle reflex and its cortical connections (Wallwork et al., 2016). Future research in this area may benefit by considering electromagnetic theories of consciousness when investigating the physiological differences between unconscious and conscious activity. Electrophysiological and imaging evidence for the correlates of consciousness may be generated by analyzing how such unconscious process interface with activity likely isomorphic to phenomenal contents.

The application of advanced imaging technologies, such as electroencephalography (EEG), functional magnetic resonance imaging (fMRI), magnetoencephalography (MEG), and diffusion tensor imaging (DTI) has yielded remarkable findings regarding the neural mechanisms underlying conscious processes by facilitating the observation and analysis of brain activity across different temporal and spatial scales. For instance, DTI and MEG studies have identified networks fundamental to the conscious experience like the thalamocortical loop (Fernández-Espejo et al., 2011; Min et al., 2020), EEG studies have provided evidence for theories of consciousness like the global workspace theory (Baars et al., 2013), and fMRI studies have revealed that brain activity underlying consciously experienced events like decisions actually precede conscious awareness and these decision outcomes can be decoded several seconds before reaching awareness (Bode et al., 2011). The incorporation of these sophisticated tools has created opportunities to further investigate the challenging relationship between neural and sensory activity and conscious experiences.

Understanding the neural basis of brain functioning requires knowledge on temporal and spatial aspects of activity (Ebrahimzadeh et al., 2022). We thus feel that the integration of dynamic reflex mechanisms with the global bioelectric architecture proposed responsible for the phenomenal representation of self and environment may be revealed and through advanced techniques such as simultaneous EEG and fMRI recording which has recently shown effectiveness in mapping multimodal functional networks (Li et al., 2022) and in other related applications (Ebrahimzadeh and Soltanian-Zadeh, 2024). Further research with these tools may additionally reveal how a unified perception of reality is manifested from a global bioelectric architecture and how information from this reality model is electrophysiologically encoded and how it is utilized by the lower-level circuits of reflexes. This architecture could be mapped by identifying its bioelectric patterns, oscillations, and networks across specific brain regions. Information encoding in specific brain areas could be decrypted by additionally including machine learning techniques. Electrophysiological decoding of visual consciousness has recently been achieved with high-quality images being produced from EEG data alone (Bai et al., 2023). To investigate the influence of this architecture over the lower-level circuits of reflexes, researchers could stimulate reflexive responses like the hand-blink reflex while participants are engaged in a conscious perceptual task. The global architecture could be tracked while measuring how it influences reflexive mechanisms in the brainstem.

The DST and other theories posit the importance of neural bioelectric oscillations as the foundation of the architecture of consciousness (Fingelkurts et al., 2010; Jerath et al., 2019). We suggest that the top-down influence of higher-level cortical knowledge of the environment on reflex mechanisms in the brainstem may be oscillatory in nature. Oscillatory synchronization between these brain areas would allow continuous integration of these conscious and unconscious processes. Given the simpler nature of reflex mechanisms relative to cortical activities, investigating such oscillatory synchronization may provide an initial means to decode the information encoded in oscillations by analyzing how these oscillations modulate reflexes. This may provide a foundation to further decode neural oscillations throughout the cortex that are prosed as isomorphic to consciousness. Research on top-down neural oscillations in reflexive mechanisms is lacking and this could be a promising avenue of research considering the perspective we have proposed. Although we have focused largely on defensive reflexes in delivering this perspective, investigation into other reflexes that utilize higher-order cognition like the social yawn reflex may yield similar insights.

**FIGURE 2:**
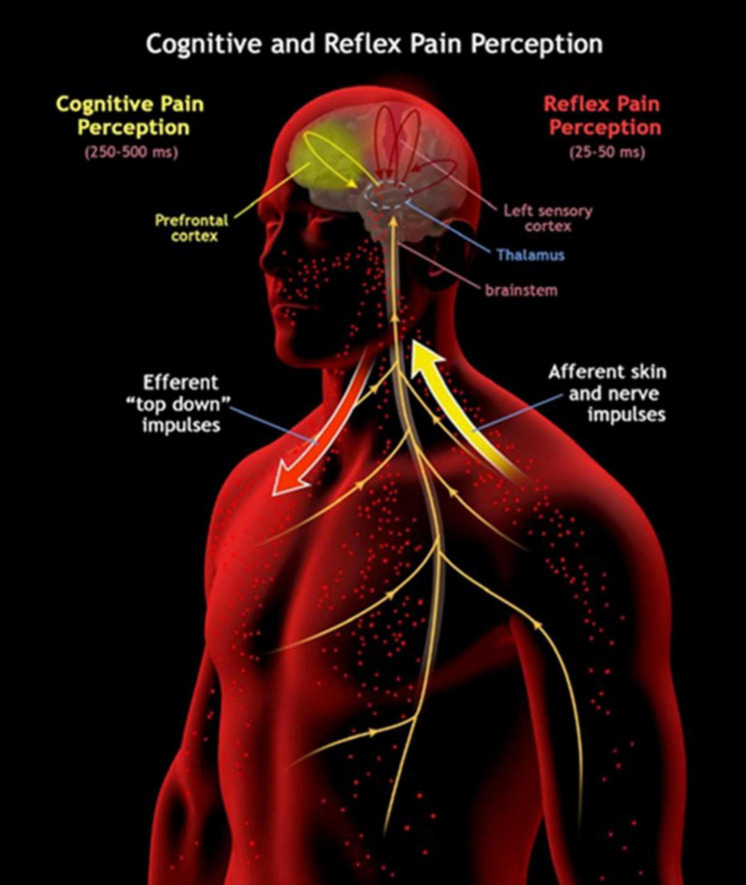
This image illustrates the two distinct pathways for pain perception in the human body: cognitive pain perception and reflex pain perception. In the cognitive pathway, higher-order brain regions such as the prefrontal cortex play a central role, resulting in a slower perception of pain spanning 250–500 ms. Both processes may involve “top-down” efferent impulses that travel from the brain to the sensory receptors themselves. The reflex pathway operates much faster, taking only 25–50 ms, and primarily involves lower-level structures like the brainstem. Here, afferent skin and nerve impulses transmit pain signals directly to these subcortical areas, enabling swift reflex responses to noxious stimuli.

Although defensive reflexes feel to us as largely unconscious, they may be better described as subconscious (Fischer and Truog, 2015) or “lightly conscious” as consciousness is proposed in some theories as quantifiable in magnitude (Tononi, 2008). Thus, in addition to an investigation into the correlates of consciousness, one into the correlates of its magnitude may also be desired. Instead of a boundary between unconscious and conscious activity, a gradient of magnitude in consciousness may be discovered with reflexes providing a small contribution to the global consciousness produced. The DST proposes that the bioelectric architecture of consciousness extends to the brainstem, and sensory and effector organs of the body given its substrate is electrical and oscillatory in nature (Jerath et al., 2019). Considering this potential for a magnitude gradient instead of a hard boundary will be essential in researching the nature of such a global bioelectrical architecture. Top-down signaling to the periphery may allow for quicker reflex responses by priming the sensory organs and their brainstem connections with sensitivity to subconsciously expected threats that may need immediate response. For instance, in regard to painful stimuli, there are separate pathways for conscious and unconscious reflexive processing (Figure 2). In a threatening situation where a painful stimulus is expected, top-down signaling from the cortex to the brainstem and to the relevant pain receptors in the body would draw cognitive attention to those receptors. If these receptors are activated by the threat, then the brainstem would be primed and informed to initiate an appropriate reflex (with further top-down influence from the cortex) as quickly as physiologically possible.

## Conclusion

We have put forth a perspective that the nature of the unconscious processes of reflexes supports the theory of an underlying cognitive structure (the default space) isomorphic to consciousness whose architecture spans the central and peripheral nervous system. A continuously existing model of the self and environment would allow rapid reflexive responses through top-down influence on the brainstem. This perspective should encourage researchers and theorists to recognize, investigate, and model the larger relationship of unconscious structured activity with activity responsible for the phenomenal mind. By revealing the nature of the need for processes responsible for reflexes to access the multimodal representation of the self and environment in computing an appropriate reflexive response, we have suggested the importance of further investigation utilizing advanced techniques like simultaneous EEG and fMRI. Investigating in detail not only the separate pathways involved in conscious and unconscious processes but also the interface between, or even a consciousness magnitude gradient among these processes may reveal neurophysiological differences between unconscious, subconscious, and conscious activity, thus potentially illuminating correlates of consciousness and its magnitude.

## Data Availability

The original contributions presented in this study are included in this article/supplementary material, further inquiries can be directed to the corresponding author.
